# The secretory function of adipose tissues in metabolic regulation

**DOI:** 10.1093/lifemeta/loae003

**Published:** 2024-01-20

**Authors:** Yang Liu, Shu-Wen Qian, Yan Tang, Qi-Qun Tang

**Affiliations:** Key Laboratory of Metabolism and Molecular Medicine of the Ministry of Education, Department of Biochemistry and Molecular Biology of School of Basic Medical Sciences and Department of Endocrinology and Metabolism of Zhongshan Hospital, Fudan University, Shanghai 200032, China; Key Laboratory of Metabolism and Molecular Medicine of the Ministry of Education, Department of Biochemistry and Molecular Biology of School of Basic Medical Sciences and Department of Endocrinology and Metabolism of Zhongshan Hospital, Fudan University, Shanghai 200032, China; Key Laboratory of Metabolism and Molecular Medicine of the Ministry of Education, Department of Biochemistry and Molecular Biology of School of Basic Medical Sciences and Department of Endocrinology and Metabolism of Zhongshan Hospital, Fudan University, Shanghai 200032, China; Key Laboratory of Metabolism and Molecular Medicine of the Ministry of Education, Department of Biochemistry and Molecular Biology of School of Basic Medical Sciences and Department of Endocrinology and Metabolism of Zhongshan Hospital, Fudan University, Shanghai 200032, China

**Keywords:** adipokines, autocrine, paracrine, endocrine, metabolic regulation

## Abstract

In addition to their pivotal roles in energy storage and expenditure, adipose tissues play a crucial part in the secretion of bioactive molecules, including peptides, lipids, metabolites, and extracellular vesicles, in response to physiological stimulation and metabolic stress. These secretory factors, through autocrine and paracrine mechanisms, regulate various processes within adipose tissues. These processes include adipogenesis, glucose and lipid metabolism, inflammation, and adaptive thermogenesis, all of which are essential for the maintenance of the balance and functionality of the adipose tissue micro-environment. A subset of these adipose-derived secretory factors can enter the circulation and target the distant tissues to regulate appetite, cognitive function, energy expenditure, insulin secretion and sensitivity, gluconeogenesis, cardiovascular remodeling, and exercise capacity. In this review, we highlight the role of adipose-derived secretory factors and their signaling pathways in modulating metabolic homeostasis. Furthermore, we delve into the alterations in both the content and secretion processes of these factors under various physiological and pathological conditions, shedding light on potential pharmacological treatment strategies for related diseases.

## Introduction

Adipose tissue is a complex organ containing mature adipocytes, preadipocytes, immune cells, sympathetic fibers, and endothelial cells, with profound effects on both physiology and pathophysiology [1–3]. Traditionally, adipocytes have been categorized into three types: white adipocytes, brown adipocytes, and beige adipocytes. In most mammals, white adipocytes are specialized for lipid storage and release, making up the bulk of adipose tissues in most animals. Excess calories are stored in white adipocytes in the form of triglycerides, and released via lipolysis during periods of fasting or thermoregulation [1, 2]. In contrast, brown adipocytes are specialized thermogenic cells able to dissipate nutritional energy in the form of heat [4, 5]. Uncoupling protein-1 (UCP1) is highly expressed in brown adipocytes, and catalyzes proton leak across the inner mitochondrial membrane, thus “uncoupling” fuel oxidation from ATP synthesis [4, 5]. This action of UCP1 mediates the thermogenic capacity of brown adipocytes. Beige adipocytes, on the other hand, are inducible brown adipocytes residing in white adipose tissue (WAT) that acquire thermogenic properties following external stimulation such as adrenergic signals, cold exposure, or exercise [6]. Human studies have demonstrated an independent correlation between the presence of brown or beige adipocytes and a reduced incidence of type 2 diabetes, dyslipidemia, and coronary artery disease [7]. Consequently, the activation of brown and beige adipocytes is considered a promising strategy for increasing systemic energy expenditure and counteracting metabolic disorders.

In addition to its role in energy storage and expenditure, adipose tissue is recognized as an indispensable endocrine organ responsible for the release of metabolites, lipids, and bioactive peptides, collectively referred to as adipokines [8, 9]. Within adipose tissues, these adipokines facilitate the intricate interplay between adipocytes and their microenvironment through autocrine and paracrine signaling to maintain tissue homeostasis. Some adipokines can be released into the circulation in an endocrine manner, serving as messengers that communicate with remote organs to orchestrate systemic energy balance. An increasing number of adipokines have been characterized, with the potent effect on fat distribution, energy expenditure, appetite and satiety, insulin secretion and sensitivity, hepatic gluconeogenesis, blood glucose, and adaptive thermogenesis [8, 9]. This review article highlights the autocrine, paracrine, and endocrine functions of adipose tissues by secreting proteins, lipids, metabolites, and extracellular vesicles (EVs), and extensively catalogs the roles in metabolic regulation.

## Proteins

### Leptin

While the endocrine role of adipose tissue has been established through observations of its secretion of adipsin/complement factor D [10] and sex steroids [11], the pivotal milestone in adipokine research could be considered the discovery of leptin [12]. Adipocytes are the primary source of circulating leptin [12]. Plasma leptin levels demonstrate a strong positive correlation with fat mass both in humans and rodents [13, 14]. The *leptin* mRNA levels are positively correlated with the size (volume) of adipocytes, as observed in size-fractioned adipocytes isolated from a single fat pad [15]. Significant variations in *leptin* gene expression among different fat depots have been extensively documented. In humans, *leptin* mRNA levels exhibit a substantial increase in subcutaneous adipose tissue compared to omental adipose tissue, irrespective of whether the individuals are lean or obese [13, 14, 16, 17]. This difference can be attributed to variations in cell size among different fat pads [17]. Interestingly, circulating leptin levels are significantly higher in females compared to males, even after adjusting for differences in body fat mass [14]. These gender-related distinctions likely result from the differences in body fat distribution between men and women, with women having relatively more subcutaneous fat than men [18]. In rodents, particularly in young adult animals, *leptin* mRNA levels are notably higher in gonadal and retroperitoneal (intra-abdominal) adipose tissues when compared to inguinal (subcutaneous) adipose tissues [19]. Leptin is also expressed in brown adipose tissue (BAT). In adult mice, *leptin* mRNA levels in BAT are much lower than in WAT [20]; while in neonatal rats, its levels in BAT are higher than inguinal WAT (iWAT) and serve as the major determinant of circulating leptin levels [21].

**Figure 1:**
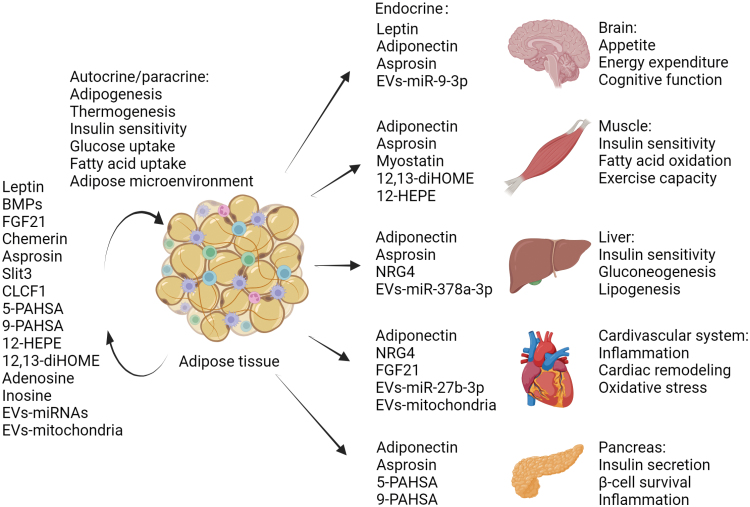
The role of adipokines in metabolic regulation (created with biorender.com).

Adipocyte-derived leptin can traverse the blood–brain barrier and enter cerebrospinal fluid via both leptin receptor (LEPR)-dependent and LEPR-independent mechanisms [22]. LEPR is known to have six distinct splicing variants, labeled from LEP-Ra to LEP-Rf. These isoforms share an identical N-terminal extracellular sequence, which is critical for leptin binding. However, they exhibit variations in their C-terminal regions, as well as in their transmembrane and intracellular domains [23]. The short LEPR isoforms LEP-Ra, LEP-Rc, LEP-Rd, and LEP-Rf, each featuring unique C-terminal sequences, and the full extent of their roles remain not completely elucidated. The LEP-Ra isoform of LEPR is thought to play a key role in the transport of leptin across the blood–brain barrier. LEP-Ra can bind leptin and mediate leptin endocytosis [24, 25]. Within hypothalamus, leptin signals through LEPR to regulate satiety, appetite, food intake, and energy homeostasis [22] (Fig. 1). Leptin is understood to primarily execute its metabolic effects through the activation of the long-form leptin receptor, LEP-Rb, in the hypothalamus [26]. LEP-Rb is the most functionally significant isoform. Two key neural populations, pro-opiomelanocortin (POMC) neurons and neuropeptide Y (NPY)/agouti gene-related peptide (AgRP) neurons in the arcuate nucleus (ARC), exert antagonistic control of appetite [27, 28]. The POMC neurons release the anorexigenic peptide α-melanocyte-stimulating hormone (α-MSH), which signals to decrease food intake by binding to melanocortin-4 receptor (MC4R) expressed by MC4R neurons in the paraventricular nucleus [27]. In contrast, NPY/AgRP neurons secrete AgRP to inhibit MC4R neurons, exhibiting an orexigenic effect [28]. LEPRs are expressed in a significant proportion of both AgRP and POMC neurons. Leptin inhibits AgRP neurons and excites POMC neurons to repress food intake [22]. During prolonged fasting, serum leptin levels decrease sharply, reflecting an adaptive physiological response to the state of starvation [29]. Beyond regulating food intake, leptin also promotes sympathetic innervation of subcutaneous WAT and BAT and enhances thermogenesis in leptin-deficient mice [30]. The effects of leptin on innervation are mediated via LEPR in AgRP and POMC neurons in the hypothalamic ARC [30]. These neurons act via brain-derived neurotropic factor-expressing neurons in the paraventricular nucleus of the hypothalamus to promote the sympathetic innervation in adipose tissues [30]. In addition to its endocrine effects, leptin also exerts its actions within adipose tissues through an autocrine mechanism, as LEPR is expressed in these tissues [31]. Leptin directly stimulates the oxidation of fatty acids by upregulating the expression of genes associated with peroxisome proliferator-activated receptor alpha (PPARα), peroxisome proliferator-activated receptor gamma coactivator 1alpha (PPARGC1α, also known as PGC-1α), and carnitine palmitoyltransferase 1 [31]. Simultaneously, it downregulates lipogenesis by reducing the expression of sterol regulatory element-binding protein 1, fatty acid synthase, and acetyl-CoA carboxylase within WAT [31]. Furthermore, leptin has been found to directly stimulate preadipocyte differentiation, primarily through the activation of PPARγ2, leading to the maturation of these preadipocytes into mature adipocytes.

Due to its appetite-reducing and energy expenditure-enhancing properties, leptin-deficient mice are massively obese. Recombinant leptin treatment provides an effective mean to reduce obesity in leptin-deficient individuals [32]. However, in the context of conventional obesity, administration of additional leptin is largely ineffective [33]. This is because obese individuals do not suffer from leptin deficiency; rather, they display higher circulating levels of leptin and exhibit central leptin resistance [34]. Hyperleptinemia is both necessary and sufficient to induce leptin resistance in both wild-type [35] and *ob/ob* mice [36]. Therefore, the approach to treating obesity by targeting leptin may require appropriate adjustment. On the one hand, combining leptin and leptin sensitizers may overcome leptin resistance and combat obesity [29]. On the other, reducing leptin levels may be useful for attenuating leptin resistance. Interestingly, Zhao *et al*. have demonstrated that pharmacological reduction of leptin levels under obese conditions, through the use of neutralizing antibodies, restores leptin sensitivity, reduces food intake, and protects mice from diet-induced obesity [37]. They propose that strategies aimed at partially reducing circulating leptin may represent a promising approach for the treatment of obesity and diabetes.

### Adiponectin

Adiponectin, a predominantly adipocyte-derived hormone, exerts pleiotropic effects on various tissues, including the liver, muscle, brain, and bone [38, 39]. Human plasma adiponectin concentrations are notably high, ranging from 2 to 20 µg/mL [40], which are around 1000-fold higher than the plasma concentrations of insulin and leptin. Adiponectin exists in diverse multimeric forms, including low molecular weight trimers, medium molecular weight hexamers, and high molecular weight (HMW) multimers, among which HMW multimers are considered the most biologically active form. The variation in adiponectin levels exhibits a marked sexual dimorphism, with females displaying higher concentrations than males, primarily due to elevated levels of HMW multimers in females [41]. Despite as an adipocyte marker, the plasma level of adiponectin is inversely with the fat mass [42], thus distinguishing it from leptin. Numerous studies have further established inverse correlations between plasma adiponectin and type 2 diabetes [43], as well as coronary artery disease [44] and myocardial infarction [45]. Circulating levels of adiponectin serve as an important indicator of adipose tissue health. Healthy fat secretes more adiponectin, while unhealthy fat, as in the case of fibrotic or inflamed adipose tissue, secretes less adiponectin [38].

Adiponectin manifests potent metabolic benefits, including insulin sensitization, anti-apoptotic properties, and anti-inflammatory/anti-fibrotic function. Both systemic and adipocyte-specific knockouts of adiponectin lead to a deterioration of insulin sensitivity [46, 47]. Consistently, injecting adiponectin improves diabetic symptoms in various obese and diabetic mouse models by decreasing triglyceride content in the muscle and liver [48, 49] (Fig. 1). The principal target organs are the liver and skeletal muscle. Adiponectin inhibits glucose production in the liver and enhances fatty acid oxidation in the skeletal muscle, at least partly through the activation of adenosine 5'-monophosphate-activated protein kinase (AMPK) and PPARα [50–52]. Moreover, adiponectin also functions in the brain, where it regulates food intake and promotes energy expenditure, resulting in body weight loss [53]. This central effect corroborates with peripheral actions to maintain the systemic energy homeostasis.

Adiponectin receptor 1 (AdipoR1) and AdipoR2 serve as the primary receptors for adiponectin and play crucial roles in regulating glucose and lipid metabolism, inflammation, and oxidative stress. AdipoR1 shows ubiquitous expression, including in the skeletal muscle and liver, while AdipoR2 expression is more restricted to the liver [54]. Obesity leads to reduced expression levels of AdipoR1 and AdipoR2 in the muscle and adipose tissues [55]. In the fasted state, there is a widespread upregulation of AdipoR1 and AdipoR2, while refeeding has the opposite effect [55]. Targeted disruption of AdipoR1 leads to the abrogation of adiponectin-induced AMPK activation, whereas AdipoR2 deficiency results in decreased activity of PPARα signaling pathways [56]. Simultaneous disruption of both AdipoR1 and AdipoR2 abolishes adiponectin’s effects, resulting in increased tissue triglyceride content, inflammation, and oxidative stress, thus leading to insulin resistance and glucose intolerance [56]. Overexpression of either AdipoR1 or AdipoR2 in hepatocytes or adipocytes results in a potent insulin-sensitizing and anti-lipotoxic phenotype [57]. Likewise, AdipoR agonist enhances insulin sensitivity and exercise endurance [58, 59], extends the shortened lifespan associated with obesity [59], and recovers nonalcoholic steatohepatitis (NASH) and related fibrosis [60]. Of note, Vailiaukaité-Brooks *et al*. have recently provided insight into the AdipoR1 and AdipoR2 structures and reported that AdipoRs exhibit ceramidase activity [61]. Ceramides are members of lipids that induce cell death, inflammation, insulin resistance, and atherosclerosis [62]. Ceramidase is responsible for converting harmful ceramides into a beneficial class of lipids, the sphinganines and sphingosines. The discovery of ceramidase activity in AdipoRs supports the earlier findings of adiponectin’s potent ceramide-reducing effects [63]. AdipoR-mediated ceramidase activity has been suggested to be related to the metabolically favorable effects of adiponectin, including the insulin-sensitizing properties in liver, and the anti-apoptotic and anti-lipotoxic effects on the cardiac myocytes and pancreatic β-cells. However, it is worth noting that AdipoR’s ceramidase activity is relatively low compared to other enzymes, even after stimulation with adiponectin. Therefore, it is suggested that AdipoRs may act on ceramides as any hydrolase and potentially possess other lipid hydrolytic activities governing downstream signal transduction [64]. Thus, additional studies are required to define the enzymatic characteristics of AdipoRs.

### Asprosin

Asprosin, a C-terminal cleavage product of fibrillin 1 (encoded by *FBN1*), is predominantly expressed and secreted by WAT, although other tissues such as cartilage and salivary glands have also been implicated in this process [65–67]. Asprosin is a fasting-induced adipokine with glucogenic and orexigenic properties, acting on the liver and orexigenic neurons, respectively [66, 68]. During fasting, elevated levels of circulating asprosin migrate to the liver, where it binds to the olfactory receptor OLFR734, activates OLFR734-coupled cyclic adenosine monophosphate (cAMP) signaling, and promotes hepatic glucose production [66, 69]. Knockout of *Olfr734* improves glucose tolerance and insulin sensitivity in obesity [69]. In addition to its glucogenic function, asprosin can cross the blood–brain barrier, directly activate orexigenic AgRP^+^ neurons, and stimulate appetite [68]. In humans, a genetic deficiency in asprosin causes neonatal progeroid syndrome characterized by low appetite and extreme leanness. This is phenocopied by mice carrying similar mutations, exhibiting lower plasma asprosin, hypophagia, and reduced body weight and fat mass, which can be fully rescued by replenishment of asprosin [68]. Protein tyrosine phosphatase receptor δ (Ptprd) is the receptor for asprosin in AgRP^+^ neurons and mediates the orexigenic effects of asprosin, loss of which results in appetite reduction and protects against diet-induced obesity [70]. A recent study identified a small-conductance calcium-activated potassium (SK) channel as the intracellular mediator for the effects of asprosin/Ptprd on AgRP neuron activation and food intake [71]. It seems likely that asprosin employs two distinct receptors for its core functions: OLFR734 receptor for asprosin-mediated hepatic glucose production and Ptprd for its orexigenic action. Moreover, asprosin can function by targeting other tissues. In the pancreas, asprosin promotes islet β-cell inflammation and apoptosis via toll-like receptor 4 and c-Jun N-terminal kinases, thereby reducing insulin secretion [72]. In the skeletal muscle, asprosin induces insulin resistance by the activation of protein kinase C-δ pathway [73]. In adipose tissues, asprosin inhibits *UCP1* expression and accelerates lipid deposition via inhibition of the nuclear factor erythroid 2-related factor 2 pathway [74]. Consistent with its metabolically unfavorable phenotype, asprosin is found pathologically elevated in individuals and rodent models with metabolic syndrome including obesity and type 2 diabetes in multiple studies [67, 68, 75–78]. Pharmacologic asprosin inhibition with neutralizing monoclonal antibody has been shown to reduce appetite and body weight and improve glycemic profile in obese mice [68, 79]. However, the underlying mechanisms driving asprosin elevation during fasting or in metabolic disorders remain unclear.

The clinical translation of secreted proteins is a crucial step in bridging the gap between laboratory research and patient care, offering new avenues for the diagnosis, treatment, and prevention of metabolic diseases. In a study involving 143 participants, grouped into three categories: normal glucose regulation (NGR), impaired glucose regulation (IGR), and newly diagnosed type 2 diabetes mellitus (nT2DM), it was observed that plasma asprosin levels were significantly higher in the IGR and nT2DM groups compared to the NGR group, especially in those with IGR [80]. Additionally, there was a direct correlation between asprosin levels and the homeostasis model assessment of insulin resistance (HOMA-IR) and an inverse correlation of asprosin levels with the homeostasis model assessment of β-cell function (HOMA-β). This clinical research suggests that asprosin could be a potent biomarker for predicting the onset of prediabetes [80].

### Chemerin

Chemerin, initially discovered as a novel retinoic acid-responsive gene in psoriatic skin lesions, has recently been identified as a novel adipokine [81, 82]. Chemerin signals through the chemokine-like receptor 1 (CMKLR1), a G protein-coupled receptor, and also the non-signaling C–C chemokine receptor-like 2 (CCRL2), both of which are expressed by a variety of cells [83, 84]. Both chemerin and CMKLR1 are expressed at high levels in WAT but low levels in BAT [85, 86] (Fig. 1). While the precise contribution of adipose tissue to circulating chemerin levels remains uncertain, human studies have demonstrated that chemerin gene expression in adipose tissues and circulating levels is positively correlated with increased body mass index (BMI) and obesity-related biomarkers [87–89].

During the differentiation of preadipocytes into adipocytes, the expressions of chemerin and CMKLR1 are dramatically increased [85, 86]. Fractionation of WAT also reveals that chemerin and CMKLR1 have significantly higher expression in mature adipocytes than stromal-vascular fraction [85, 86]. A recent study has revealed an inhibitory function of chemerin in adaptive thermogenesis, which depends on its autocrine and paracrine actions. Cold exposure results in decreased expression of chemerin and CMKLR1 in iWAT [86]. The chemerin-CMKLR1 axis inhibits the production of interleukin (IL)-33 in adipocytes, which is a critical upstream cytokine to initiate type 2 immune responses, facilitating the formation of beige adipocytes [90]. Lack of chemerin or adipocytic CMKLR1 activates cold-induced thermogenic beige fat, enhances energy expenditure, and exhibits metabolically favorable effects [86]. Conflicting results have emerged regarding the role of chemerin signal in adipogenesis and whole-body metabolism. Some observations show that activation of chemerin-CMKLR1 axis facilitates the proliferation and differentiation of preadipocytes, as well as the angiogenesis in fat pad [91]. Inactivation of chemerin signal inhibits the adipogenesis process [92]. Disruption of CMKLR1 *in vivo* reduces the body mass and fat deposition of mice and improves the glucose tolerance [86, 93]. Treatment with chemerin exacerbates obesity-associated glucose intolerance in *ob/ob* mice, *db/db* mice, and high-fat diet (HFD)-fed mice [94]. In contrast, other studies have shown a protective effect of chemerin signal in metabolic homeostasis. These studies demonstrate that *Cmklr1* knockout mice display mild obesity. They found that the differentiation of preadipocytes into adipocytes is not affected by loss of CMKLR1 [95]. Also, mice overexpressing chemerin specifically in the liver exhibit improved glucose tolerance [96]. Overall, the role of the chemerin signal in metabolic regulation has remained controversial. It is worthwhile to determine whether sex, diet, genetic background, and sanitary status of animals influence the results. Employing more sophisticated mouse models might be essential for elucidating the precise effects of this signaling axis on metabolic homeostasis. This could involve strategies such as carefully timed and cell type-specific overexpression or deletion of chemerin or its receptors.

### NRG4

BAT has long been thought to have only one major function—non-shivering thermogenesis through UCP1. This notion was challenged by an intriguing finding that although mice lacking *UCP1* exhibit cold intolerance, they are resistant to diet-induced obesity at ambient temperatures and become predisposed to weight gain only at thermoneutrality [97–99]. In contrast, mice lacking BAT due to either surgical resection or genetic ablation exhibit not only cold intolerance but also a greater susceptibility to obesity [100, 101]. The observation that ablation of BAT causes more severe metabolic disorders compared to *UCP1* deficiency implies that BAT may influence energy balance through mechanisms beyond thermogenic activity, possibly including endocrine functions. This notion gains further support from the discovery of brown adipokines, also referred to as batokines. Apart from common adipokines shared with white adipocytes, such as adiponectin and leptin, brown adipocytes highly secrete some batokines such as neuregulin 4 (NRG4), bone morphogenic protein 8B (BMP8B), myostatin, adipose-secreted signaling protein (Adissp), and cardiotrophin-like cytokine factor 1 (CLCF1). These brown adipokines have the capacity to regulate BAT thermogenesis through autocrine and paracrine effects, as well as influence metabolic homeostasis in distant tissues through endocrine function (Fig. 1). For instance, thermoneutrality promotes the secretion of myostatin by brown adipocytes, which acts as an inhibitor for muscle function and results in decreased exercise capacity [102]. Adissp and CLCF1 are recently identified batokines [103, 104]. Adissp is a positive regulator for thermogenesis. Transgenic expression of *Adissp* enhances thermogenesis, improves glucose homeostasis, and protects against diet-induced obesity [104]. On the contrary, CLCF1 is an inhibitor of thermogenesis, which is downregulated by thermogenic stimuli and upregulated in obesity [103]. Transgenic expression of *CLCF1* results in impaired mitochondrial biogenesis in brown fat and renders mice more prone to develop metabolic disorders.

NRG4, one of the extensively studied batokines, is a member of the epidermal growth factor family of extracellular ligands. NRG4 is highly enriched in brown fat, with lower levels found in WAT and other tissues. In adipose tissues, NRG4 is mainly expressed in mature adipocytes, and its expression is elevated by cold exposure. Lin group has identified NRG4 as a batokine that targets the liver and maintains metabolic homeostasis in the liver through its endocrine activity. They demonstrate that NRG4 derived from brown fat represses hepatic lipogenesis through the signaling of human epidermal growth factor receptor 3 (ErbB3, HER3) and ErbB4 (HER4), ultimately leading to the inhibition of nonalcoholic fatty liver disease (NAFLD) [105]. *NRG4*-deficient mice are susceptible to obesity, insulin resistance, and hepatic steatosis. In addition to the suppression of hepatosteatosis, Lin group found that NRG4 inhibits the progression from hepatic steatosis to NASH by protecting hepatocytes from stress-induced cell injury [106]. Also, they demonstrated that NRG4 represses NASH-related hepatocellular carcinoma (HCC) by restraining tumor-prone liver microenvironment. *NRG4* deficiency exacerbates NASH-associated induction of intrahepatic CD8^+^ T cell exhaustion and renders mice more prone to development of HCC. Recombinant NRG4-Fc fusion protein exhibits remarkable potency in suppressing HCC and prolongs survival of treated mice [107]. NRG4 also has a significant impact on arteries (Fig. 1). It is recently reported that NRG4 decreases apoptosis, inflammation, and adhesion responses in vascular endothelial cells. BAT-specific *NRG4* deficiency accelerates vascular inflammation, adhesion responses, endothelial dysfunction, apoptosis, and atherosclerosis in mice [108]. While endocrine signal by NRG4 has been well studied, autocrine or paracrine function of NRG4 remains poorly understood. Despite its abundant expression in BAT, the role of NRG4 in cold-stimulated BAT thermogenesis appears to be dispensable [105]. Wild-type and *NRG4*-deficient mice have similar rectal body temperature and thermogenic gene expression in BAT [105]. In contrast, NRG4 seems to be required for thermogenic capacity of WAT [109]. *NRG4* deficiency attenuates cold-induced iWAT browning and *UCP1* expression [109]. Emerging evidence suggests that NRG4 promotes sympathetic neuron axonal growth and branching *in vitro* [110, 111], presenting the possibility that NRG4 may enhance browning process by promoting sympathetic innervation of WAT. However, further validation is needed to determine whether NRG4 genuinely mediates adipocyte-nerve communication.

The expression levels of NRG4 in adipose tissues are obviously decreased in both rodent and human obesity and negatively correlated with the liver fat content [105]. Pro-inflammatory factors like tumor necrosis factor α (TNF-α) and IL-1β reduce NRG4 expression in adipocytes, likely contributing to the reduced NRG4 in obesity [105]. Consistently, a cross-sectional study reported significantly lower serum NRG4 levels in patients with metabolic syndrome compared to normal controls. Circulating NRG4 levels are inversely correlated with waist circumference and BMI [112]. By whole-exome sequencing and exome genotyping of obesity, a recent study has identified two rare missense mutations in NRG4: Nrg4 E47Q and Nrg4 R44H [113]. Nrg4 E47Q is shown to enhance the protective effects of NRG4 against NAFLD, whereas Nrg4 R44H lacks this function [113]. Mechanistically, Nrg4 E47Q has a higher affinity to bind ErbB4 than WT NRG4, which activates ErbB4 to negatively regulate *de novo* lipogenesis in hepatocyte, while Nrg4 R44H loses the binding affinity with ErbB4 [113]. This indicates that genetic variation in the population generates an aberrant function of NRG4, which could serve as either a risk factor or a protective factor for NAFLD and associated metabolic disorders. Overall, NRG4 is a brown fat-derived endocrine checkpoint that exhibits potently protective effects on metabolic disorders in animal models, including insulin resistance, hepatosteatosis, NASH, HCC, and atherosclerosis. The potential application of NRG4 in the intervention of metabolic syndrome is worth expecting.

### BMPs

The BMP family, which belongs to the transforming growth factor superfamily, plays pivotal roles in the development and maintenance of numerous tissues [114]. These proteins transmit signals by forming complexes with one of seven distinct type I receptors, known as activin receptor-like kinases 1–7 (ALK1–7), along with one of three different type II receptors: BMP receptor 2 (BMPR2), activin receptor (ACVR) 2a, and ACVR2b [115]. BMP2 and BMP4 are expressed in both WAT and BAT, with BMP4 expression positively correlated with adiposity and adipocyte size [116, 117]. Both BMP2 and BMP4 promote the commitment and differentiation of adipose tissue stromal cells to the adipogenic lineage [118–120]. Mice with adipocyte-specific overexpression of *Bmp4* exhibit reduced WAT mass and increased BAT weight [117, 121]. This overexpression also triggers augmented WAT angiogenesis and browning, while causing a phenomenon of whitening in BAT. Notably, this leads to an overall increase in energy expenditure and an enhancement in glucose tolerance and insulin sensitivity [117, 121]. Interestingly, specific knockout of *Bmp4* in adipocytes produces contrasting effects, leading to elevated WAT and BAT masses, decreased WAT angiogenesis, BAT whitening, and disruptions in glucose tolerance and insulin sensitivity [117, 121]. Similar outcomes are observed when *Bmp4* is overexpressed using viral vectors, either systemically or locally in BAT [122, 123]. In adipose tissues, BMP4 also induces the activation of M2 macrophages to facilitate the browning of WAT [124]. The mechanism by which adipose tissue macrophages promote browning process is controversial. Nguyen *et al*. demonstrated that M2 macrophages sustain adaptive thermogenesis in adipose tissues by producing catecholamines [125]. In contrast, Fischer *et al*. contended that adipose tissue M2 macrophages do not synthesize catecholamines, thereby proposing the involvement of other unknown mediators responsible for their promotive effect on adaptive thermogenesis [126]. Wang *et al*. appeared to uncover such a mediator. They identified Slit3 as an M2 macrophage-secreted cytokine that increases sympathetic activity to enhance browning process [127]. Additionally, endocrine function of BMP4 plays a crucial role in systemic homeostasis. For example, adipose tissue BMP4 exhibits endocrine function through targeting ovary, where it inhibits androgen synthesis and promotes estrogen production [128]. Adipocyte-specific overexpression of *Bmp4* in mice protects against hyperandrogenemia and polycystic ovary syndrome [128].

BMP7 stands out as the BMP most notably associated with brown adipogenesis. Tseng *et al*. demonstrated that BMP7 effectively stimulates the differentiation of cultured brown preadipocytes, displaying greater efficiency in enhancing the expression of *UCP1* and mitochondrial biogenesis compared to other BMPs [129]. Additionally, in *Bmp7* knockout mice, there is a remarkable reduction of 50% to 70% in interscapular BAT mass when compared to their wild-type littermates [129]. C57BL6/J mice treated with BMP7 via subcutaneous osmotic minipumps exhibit an increase in BAT volume, along with an elevation in *UCP1* expression and energy expenditure [130]. These mice also display browning of WAT accompanied by diminished WAT mass [130].

BMP8B has been implicated in regulating energy expenditure [110, 131–133], NASH development [134], germline cell proliferation and maturation [135, 136], and cancer [137]. Antonio Vidal-Puig group identified BMP8B as a batokine [110, 133]. They observed that *Bmp8b* mRNA expression is highest in BAT and testis, and is also significantly expressed in the brain, while there is almost no expression in iWAT, epididymal WAT, liver, and muscle [133]. Fractionation of BAT showed that *Bmp8b* expression is restricted to the mature adipocyte population, which is in stark contrast to some other BMPs like BMP2, BMP4, and BMP5, all of which are mainly enriched in stromal-vascular cells [133]. *Bmp8b* expression in BAT displays a robust increase following HFD feeding and cold stimulation. Thermogenic stimuli, including β3-adrenoceptor agonist CL316243 and thyroid hormone, can drive the expression of *Bmp8b* in BAT [133]. Consistently, a study in human neonates showed that serum BMP8B levels are increased with a single short-term cold stimulus [132]. Besides, estrogens can induce *Bmp8b* expression; therefore, *Bmp8b* expression in BAT exhibits sex difference, with higher expression in female mice than males [138].

Antonio Vidal-Puig group has elucidated that BMP8B acts both in peripheral (adipose tissues) and central (hypothalamus) to promote BAT thermogenesis. In adipose tissues, BMP8B on one hand enhances p38 mitogen-activated protein kinase (MAPK)/cAMP response element-binding protein (CREB) signaling and promotes lipase activity in mature adipocytes [133]; on the other hand, it promotes sympathetic innervation and vascularization in adipose tissues through paracrine action [110], thereby increasing the thermogenic capacity of BAT and inducing browning process of WAT. *Bmp8b* is also expressed in the hypothalamus, where it regulates AMPK activity and increases sympathetic activation of BAT [133]. However, it is important to note that the aforementioned thermogenic action of BMP8B was observed only in female rodents, suggesting that the thermogenic effect of BMP8B is sexually dimorphic [133, 139]. Consistently, Martins *et al*. found that intracerebroventricular injection of BMP8B promotes BAT thermogenesis in female rats, but has no effect in males and ovariectomized (OVX) females [139]. Estradiol replacement restores the response to BMP8B in OVX rats [139], but the interplay between BMP8B and estradiol during thermogenic activation is still unknown. As a result, female mice deficient in BMP8B display impaired thermogenesis and reduced metabolic rate, causing obesity, whereas male mice lack of BMP8B exhibit no phenotype in energy metabolism [133]. Martins *et al*. also clarified the mechanism underlying the regulation of central BMP8B in thermogenesis [139]. BMP8B inhibits AMPK activity in the ventromedial nucleus of the hypothalamus (VMH) and subsequently increases orexin signaling in the lateral hypothalamic area, which in turn activates sympathetic nervous system outflow to induce BAT thermogenesis [139]. Like many metabolically favorable hormones, obesity also induces resistance to central action of BMP8B [131]. Central administration of BMP8B could not activate thermogenic adipocytes and promote weight loss in HFD-fed female rats. The underlying mechanism is that (i) BMP8B treatment induces a decrease in BMP receptors in VMH of obesity; (ii) BMP8B loses the ability to inhibit AMPK [131]. Overall, BMP8B, derived from both adipocytes and hypothalamus, functions through autocrine and paracrine signaling, synergistically activating thermogenic adipocytes and protecting against diet-induced obesity in females. However, as an adipokine originated from brown and beige adipocytes, the endocrine action of BMP8B is poorly characterized and awaits further clarification.

### Fibroblast growth factor 21 (FGF21)

FGF21 is a peptide hormone known for its metabolic benefits, such as weight loss in obesity and improved hyperglycemia [140]. The signaling transduction of FGF21 requires FGF receptor (FGFR) and the coreceptor β-klotho (KLB) [141]. While the liver is the primary source of circulating FGF21 in most conditions, thermogenic adipocytes can also generate and secrete significant amounts of FGF21 in response to cold or adrenergic stimulation [142–144]. The expression of *FGF21* and its release from BAT are regulated by cAMP-protein kinase A (PKA)-p38 MAPK-activating transcription factor 2 axis [144]. FGF21 functions through autocrine and paracrine actions to promote the browning of WAT (Fig. 1). One explanation for the effect of FGF21 is that it directly upregulates PGC-1α, thereby inducing the browning process [145]. Moreover, research by Xu’s group demonstrates that the activation of type 2 immunity in WAT mediates FGF21’s effect. They found that FGF21 acts on adipocytes in an autocrine manner to promote the expression and secretion of C–C motif chemokine ligand 11 (CCL11), which drives the recruitment of eosinophils in WAT, leading to increases in accumulation of M2 macrophages and promoting adipocyte precursors into beige adipocytes [146]. In addition to inducing browning, FGF21 enhances mitochondrial oxidation, promotes insulin-independent glucose uptake, and induces secretion of adiponectin in adipocytes, which in turn mediates the glucose-lowering and insulin-sensitizing effects of FGF21 [147–149]. However, the question of whether BAT-derived FGF21 contributes to systemic FGF21 levels remains controversial. Abu-Odeh *et al*. showed that adipocyte-secreted FGF21 does not enter circulation [142]. They found that an adrenergic-dependent increase in circulating FGF21 occurs through an indirect mechanism in which fatty acids released by adipocyte lipolysis subsequently activate hepatic PPARα and increase *FGF21* expression. This demonstrates that the increased circulating FGF21 under adrenergic induction is derived exclusively from the liver, not BAT [142]. In contrast, some other studies have implicated adipocytes in systemic FGF21. Ruan *et al*. showed that surgical depletion of BAT decreases serum FGF21 levels [150]. They also found that BAT-derived FGF21 targets heart and protects against hypertensive cardiac remodeling [150]. Additionally, certain experimental inventions, such as genetic disruption of the *UCP1* gene [151] or BAT transplantation [152], can cause BAT to become a significant source of secreted FGF21. Nevertheless, it seems likely that the function of autocrine FGF21 differs from the circulating FGF21. For example, Abu-Odeh *et al*. showed that adipocyte-secreted FGF21 contributes to browning of white adipocytes, but circulating FGF21 is dispensable for the browning process. Further studies are needed to distinguish the functions of FGF21 as an autocrine and endocrine factor.

The interactions among key secreted proteins are pivotal in the regulation of metabolism, encompassing an intricate network of synergistic effects. These cytokines facilitate critical communication across various organs and tissues. FGF21 effectively prevents TNF-α from hindering adiponectin secretion. When FGF21 is incubated with primary murine adipocytes, it significantly enhances the secretion of adiponectin into the medium, demonstrating even greater potency than the PPARγ agonist rosiglitazone [148]. These data suggest that FGF21 is a highly effective regulator of adiponectin secretion and its ability to modulate blood sugar levels and enhance insulin sensitivity is significantly reliant on the presence of adiponectin [148]. This finding highlights FGF21–adiponectin axis’s promising role in metabolic-homeostasis regulation.

### Lipokines

In addition to proteins or peptides, adipose tissues can release bioactive lipids, known as lipokines. The concept of lipokine was first proposed by Cao *et al*. in 2008 to describe a class of lipid hormones connecting adipose tissues to systemic metabolism [153]. Over the past 15 years, there has been significant progress in understanding adipose-secreted lipokines, including C16:1n7-palmitoleate [153], lysophosphatidic acid [154], alkyl ether lipids [155], fatty acid esters of hydroxyl FAs (FAHFAs), palmitic acid hydroxystearic acids (PAHSAs) [156], and oxidized lipid metabolites derived from polyunsaturated fatty acids [157]. These lipokines systemically act as hormonal regulators and signaling mediators that are involved in regulating nutrient utilization, adaptive thermogenesis, and systemic metabolism.

Since the discovery of branched FAHFAs in 2014 [156], various FAHFA families have been identified in mouse WAT. The FAHFA isomers differ in the location of the branched ester on the hydroxy fatty acid, and they exhibit distinct biological activities [156]. Among these, 5-PAHSA and 9-PAHSA, the most extensively studied FAHFA subfamily, have shown anti-diabetic and anti-inflammatory effects in both humans and rodents [156]. Low circulating 5-PAHSA levels are associated with insulin resistance [156]. Both acute and chronic administration of 5-PAHSA and 9-PAHSA improve insulin sensitivity and glucose tolerance [156, 158]. It is suggested that 9-PAHSA and possibly 5-PAHSA exert their effects through G protein-coupled receptor 40 (GPR40) and GPR120, which are expressed in various cell types [159]. 5-PAHSA and 9-PAHSA may directly act on adipose tissue stromal cells to promote adipogenic differentiation, and in adipocytes to increase insulin-stimulated glucose uptake, in β-cells to promote glucose-stimulated insulin secretion, in l-cells to enhance glucagon-like peptide-1 (GLP-1) secretion, and in macrophages to decrease activation and pro-inflammatory cytokine release [156, 160, 161] (Fig. 1). Of note, Patel *et al*. recently identified adipose triglyceride lipase (ATGL) as the biosynthetic enzyme for FAHFAs [162], which has resolved the longstanding question regarding FAHFA synthesis process. However, despite refutations citing non-reproducible results were due to different experimental methodologies and cell culture systems [163], controversial studies have concluded that both 5-PAHSA and 9-PAHSA do not influence insulin release or glucose uptake in associated cells *in vitro*. Additionally, these compounds were found ineffective in ameliorating metabolic markers in diet-induced obesity mice when tested *in vivo* [164].

The 12,13-dihydroxy-9Z-octadecenoic acid (12,13-diHOME) is a recently identified lipokine secreted by BAT, which is an oxidized linoleic acid metabolite [165]. Exposure to cold and exercise increases the systemic concentration of 12,13-diHOME [157, 166]. While not the unique origin for basal serum 12,13-diHOME, BAT appears to be the primary contributor to the elevation of circulating 12,13-diHOME induced by cold and exercise [157, 166]. Cold-induced 12,13-diHOME is acutely produced in BAT through the upregulation of epoxide hydrolase 1 (*Ephx1*) and *Ephx2*, genes related to its biosynthesis, and it activates BAT thermogenesis through both autocrine and paracrine action [157]. Specifically, 12,13-diHOME stimulates the translocation of fatty acid transporters, fatty acid transport protein 1 and cluster of differentiation 36, to the cell membrane, which mediates fatty acid absorption in BAT and facilitates fuel supply for thermogenesis. Mice treated with 12,13-diHOME exhibit increased BAT-specific lipid uptake and enhanced cold tolerance, which results in decreased levels of serum triglyceride [157]. These findings indicate a complex process of fuel consumption and refueling during cold exposure. In cold conditions, fatty acids in brown adipocytes serve both as fuels to be oxidized for thermogenesis and as substrates for 12,13-diHOME biosynthesis. As 12,13-diHOME stimulates lipid uptake in brown adipocytes, cellular fuel consumption is coupled with a potent refueling signal, which promotes lipid update in BAT and forms a self-reinforcing and beneficial cycle, particularly in the context of obesity [157]. Several independent cohort studies have shown that plasma concentrations of 12,13-diHOME are negatively associated with BMI, total fat mass, HOMA-IR score, and plasma triacylglycerol levels [157, 166, 167].

Apart from cold stimulation, exercise causes a significant increase in circulating 12,13-diHOME in both humans and rodents [166]. Surgical removal of BAT abolishes the exercise-induced increase in 12,13-diHOME, suggesting that BAT is the tissue source for its elevation during exercise [166]. Under conditions of exercise training, 12,13-diHOME released by BAT functions in an endocrine manner, promotes fatty acid uptake and oxidation in the skeleton muscle, and increases mitochondrial respiration in the muscle [166]. This endocrine role of 12,13-diHOME is further validated by the observation that BAT-derived 12,13-diHOME promotes respiration in cardiomyocytes and enhances cardiac function [168]. In summary, 12,13-diHOME serves as a batokine with both autocrine and endocrine action, and renders BAT and muscle as metabolic sink for fatty acid during cold and exercise, respectively, which suggests potential applications for the treatment of hyperlipidemia. It is worth noting that 12,13-diHOME-induced fatty acid absorption is tissue specific, as it has no effect on tissues like WAT, liver, and heart. The mechanism underlying this specificity remains unknown and requires further investigation, as well as the possible explanation for the translocation of fatty acid transporter regulated by 12,13-diHOME.

Another BAT-derived lipokine, 12-hydroxyeicosapentaenoic acid (12-HEPE), is synthesized from polyunsaturated fatty acid by 12-lipoxygenases (12-LOXs). Both cold exposure and β3-adrenergic stimulation induce an increase in circulating 12-HEPE levels in both humans and rodents [169]. BAT is the main source for the increased 12-HEPE levels during cold stimulation [169]. 12-HEPE promotes glucose uptake into adipocytes and skeletal muscle by activating an insulin-like intracellular signaling pathway [169]. Deletion of 12-LOX in brown adipocytes inhibits 12-HEPE production, leading to impaired glucose uptake and metabolism, ultimately resulting in cold intolerance [169]. Notably, plasma 12-HEPE levels are negatively related to obesity and HOMA-IR [169]. Overall, an increasing number of BAT-derived lipokines have been identified, each exhibiting metabolically regulatory function. These lipokines potently regulate nutrient uptake through autocrine and endocrine function (Fig. 1). Further investigations are needed to uncover the precise mechanisms underlying how these lipokines induce fatty acid and glucose uptake. Additionally, identifying potential receptors for these lipokines will enhance our understanding of their actions and contribute to the development of lipokine-based therapies for the treatment of metabolic diseases.

### Nucleosides

Nucleosides are biologically important molecules with various roles, including nucleic acid synthesis, energy metabolism, and acting as signaling molecules. One such nucleoside, adenosine, is a ubiquitous endogenous autacoid that exerts its effects through adenosine A_1_ and A_3_ receptors, mediated by Gi, or via A_2A_ and A_2B_ receptors, utilizing Gs signaling pathways [170]. Adenosine can be secreted in BAT through two major mechanisms: breakdown of ATP released from sympathetic nerves [171] and produced within brown adipocytes [172, 173]. Adenosine activates lipolysis in both human and murine brown adipocytes at low nanomolar concentrations [173]. Among adenosine receptors, adenosine A_2A_ receptor is the most abundant in human and murine BAT. Genetic loss or pharmacological blockade of A_2A_ receptors in mice causes a decrease in BAT-dependent thermogenesis [173]. Treatment with A_2A_ agonists significantly promotes the browning of WAT, increases energy expenditure, and protects diet-induced obesity [173]. Moreover, adenosine is also a major precursor of ATP. A recent study demonstrated that ATP enhances lipolysis in adipocytes by facilitating the nascent protein synthesis of ATGL, thereby promoting adaptive thermogenesis [174]. It is worth noting that the role of adenosine in brown adipocytes exhibits species-specific variations. In contrast to findings in humans and mice, adenosine suppresses lipolysis in brown adipocytes from hamsters and rats, reducing their sensitivity to catecholamines [172, 175–177].

Adipose tissues display a remarkable ability to adapt to changes in nutritional and metabolic states, revealing a plasticity of both proliferation and apoptosis. In the context of obesity or aging, BAT experiences continuous apoptosis. Niemann *et al*. demonstrated that apoptotic brown adipocytes release a specific pattern of metabolites, with purine metabolites being highly enriched [178]. When healthy brown adipocytes are incubated with the supernatant of apoptotic brown fat cells, it results in a significant increase in the expression of thermogenic marker genes [178]. Apoptotic brown adipocytes secrete inosine to activate the thermogenic capacity of neighboring cells, effectively replacing the apoptotic cells. Inosine signals via the receptors A_2A_ and A_2B_ in thermogenic adipocytes to activate the cAMP/PKA/p38 pathway. Treatment of mice with inosine increases BAT-dependent energy expenditure and induces browning of WAT, thereby protecting against diet-induced obesity [178].

### EVs

In addition to the classical adipokines, adipose tissues can also produce and secrete EVs [179]. Exosomes are a subset of EVs with diameters ranging from 30–150 nm. They are released following the fusion of intermediate endocytic compartment, multivesicular bodies (MVBs), with the plasma membrane. These exosomes carry diverse bioactive cargos, including proteins, nucleic acids, and lipids derived from parent cells [179]. miRNAs are 19–22-nucleotide-long non-coding RNAs that function as regulators of translation. Many miRNAs exist in tissues and circulation, and a large fraction of these are found in exosomes [180]. Importantly, it has been established that miRNAs are not randomly incorporated into exosomes; rather, they possess sorting sequences that determine their exosome secretion or cellular retention [181]. Interestingly, different cell types make preferential use of specific sorting sequences, thus defining the exosomal miRNA profile of each cell type [181].

Both in humans and mice, adipose tissue serves as the primary source of circulating exosomal miRNAs [182]. The amount of miRNA in WAT declines with age, due to a decrease in the miRNA-processing enzyme Dicer [180]. Adipocyte-specific Dicer knockout mice (ADicerKO) exhibit impaired miRNA processing in adipose tissue, resulting in a substantial decrease in the levels of circulating exosomal miRNAs [182]. Transplantation of both WAT and BAT into ADicerKO mice restores the levels of numerous circulating miRNAs [182]. Lipodystrophy patients, featured by defects in fat tissues, also display decreased circulating exosomal miRNA levels [182]. These findings collectively suggest that adipose tissue is an important source of circulating miRNA, particularly those present in exosomes. Specific miRNAs derived from adipose tissues, such as miR-222, miR-27a, and miR-130b, which are increased in the serum of obese humans and mice, have been shown to induce insulin resistance in the skeletal muscle or liver respectively by targeting insulin receptor substrate 1 [183], PPARγ [184], and PGC-1α [185]. Exosomal miR-27b-3p secreted by visceral adipocytes contributes to endothelial inflammation and atherogenesis [186]. Moreover, cold exposure facilitates the selective packaging of miR-378a-3p into EVs and delivery into the liver. BAT-derived miR-378a-3p enhances gluconeogenesis by targeting the catalytic subunit p110α of phosphatidylinositol 3-kinase (PI3K), contributing to the cold-induced elevation in hepatic gluconeogenesis [187]. Wang *et al*. have recently demonstrated that adipose-derived EVs and their cargo miRNAs mediate inter-organ communication between adipose tissue and the brain. These EVs can be transferred into the brain in a membrane protein-dependent manner and are enriched in neurons, especially in the hippocampus [188]. Adipose-derived EV miRNAs such as miR-9-3p, which is increased in obesity and type 2 diabetes, mediate synaptic damage and lead to diabetes-related cognitive impairment [188] (Fig. 1). Adipocyte-derived exosomes can also function through a paracrine manner. Adipocyte-secreted exosomal microRNA-34a can be transported into macrophages within adipose tissues, suppressing M2 polarization by repressing the expression of Krüppel-like factor 4, thereby promoting metabolic inflammation and insulin resistance [189]. Adipocyte-specific miR-34a–KO mice are resistant to obesity-induced insulin resistance, glucose intolerance, and systemic inflammation [189]. In addition, adipocyte-derived exosomal miR-155 targets adipose tissue-resident macrophages, promotes signal transducer and activator of transcription 1 (STAT1) signaling, and suppresses STAT6 signaling, resulting in M1 macrophage polarization and subsequent insulin resistance [190]. These studies together demonstrate that adipocyte-derived miRNAs play a crucial role in metabolic regulation in neighboring cells and distal organs, with both beneficial and deleterious metabolic effects depending on the particular miRNA and its target.

Other cell types in adipose tissues also secrete exosomal miRNAs that regulate metabolic homeostasis. Exosomes originating from adipose-derived stem cells (ADSCs) alternatively activate M2 macrophage polarization, attenuate inflammation, and promote the browning process in WAT [191]. Treatment of obese mice with ADSC-derived exosomes improves insulin sensitivity and alleviates obesity and hepatic steatosis [191]. Besides, exosomes secreted by adipose tissue macrophages can transfer miRNAs to distant target organs [192]. Treatment of obese mice with lean adipose tissue macrophage exosomes improves insulin resistance; while treating lean mice with adipose tissue macrophage exosomes from obese mice causes insulin resistance [192]. miR-155 is one of the miRNAs that is overexpressed in obese adipose tissue macrophages and can induce insulin resistance in adipose tissues, skeletal muscle, and liver by downregulation of PPARγ expression [192]. In contrast, miR-690, secreted by M2-polarized macrophages, improves insulin sensitivity and glucose tolerance by targeting NAD^+^ kinase in adipocytes and hepatocytes [193]. These findings suggest that adipose tissue-resident macrophages secrete exosomal miRNAs with the potential to regulate systemic insulin sensitivity and may serve as a source of therapeutic miRNAs for the treatment of obesity-related disorders.

Furthermore, EVs carry abundant proteins that play a crucial role in metabolic regulation in recipient cells. Analysis of the protein profiles of EVs has confirmed that EVs from rodents are enriched in proteins and enzymes involved in the metabolism and transport of lipids, such as caveolin 1, fatty acid synthase, and lipoprotein lipase [194, 195]. In particular, enzymes related to *de novo* lipogenesis, including acetyl-CoA carboxylase, glucose‑6‑phosphate dehydrogenase, and fatty acid synthase, are selectively enriched in EVs from 3T3-L1 adipocytes. These proteins in EVs promote lipid accumulation in recipient adipocytes and preadipocytes [196]. Exosomes derived from ADSCs carry active STAT3 as a protein cargo, which can be transferred into macrophages to induce anti-inflammatory M2 phenotypes by transactivating arginase-1 [191]. These exosomes reduce inflammation and promote browning of WAT by delivering STAT3 to the macrophages [191]. Beyond non-coding RNAs and proteins, metabolites are important cargos in EVs. For example, α-ketoglutarate is enriched in adipose-derived exosomes upon melatonin treatment, which exhibits anti-inflammatory effects [197]. By transporting α‐ketoglutarate to macrophages, the adipocyte-derived exosomes promote anti-inflammatory M2 polarization and thus attenuate the adipose inflammation in obesity [197]. In addition, adipocytes not only hydrolyze triglycerides to release fatty acids extracellularly but also transport intact triglyceride molecules via adipocyte EVs [198]. These EVs are absorbed by adipose tissue macrophages, providing a source of lipids for these local cells [198]. This process operates independently from classical lipolysis. Obese mice release over twice the amount of lipids per day through exosomes compared to lean mice. Simultaneously, adipose EVs encourage the differentiation of bone marrow progenitor cells into adipose tissue macrophages [198]. Research has also demonstrated that EVs derived from adipose tissue possess a distinctive lipid profile, which is rich in ceramides, sphingolipids, and phosphatidylglycerols, in contrast to the lipid composition of adipose tissue itself [199]. Furthermore, obesity has been found to modify the lipid profile of adipose tissue-originated EVs [199]. The alterations in the composition of metabolites within EVs and the related functions of these metabolites under physiological and pathological conditions warrant further investigation.

Interestingly, emerging evidence suggests that EVs may serve as an alternative pathway for cellular quality control. Several studies have identified mitochondrial components within the cargo of EVs [200–202]. Cells can eject damaged mitochondria through EVs. Crewe *et al*. have shown that adipocytes respond to mitochondrial stress by promptly and robustly releasing small EVs, which contain respiration-competent, but oxidatively damaged mitochondrial particles [201]. These EVs enter circulation and are taken up by cardiomyocytes, where they induce transient mitochondrial dysfunction of the host network and lead to the production of ROS [201]. It is important to note that this process is not pathological; instead, it triggers compensatory antioxidant signaling in the heart that protects cardiomyocytes from acute oxidative stress. Thus, a single injection of small EVs from energetically stressed adipocytes limits cardiac ischemia/reperfusion injury in mice [201]. Additionally, brown adipocytes also release damaged mitochondrial components through EVs under thermogenic stress [202]. When re-uptaken by parental brown adipocytes, these EVs containing damaged mitochondria inhibit the levels of mitochondrial proteins, including UCP1. This has a detrimental effect on brown fat metabolism and exerts a negative autocrine impact on brown adipocyte thermogenesis [202]. BAT-resident macrophages are involved in clearing the mitochondrial components ejected from brown adipocytes, thereby maintaining the thermogenic program of BAT. Depletion of macrophages results in the accumulation of extracellular mitochondrial vesicles in BAT, impairing the thermogenic activity during cold exposure [202].

## Perspective

Adipose tissues comprise adipocytes, precursor cells, fibroblasts, endothelial cells, and immune cells, all contributing to the release of bioactive peptides, metabolites, lipids, and EVs. These adipose tissue-derived factors function through autocrine or paracrine within adipose tissues to regulate processes such as adipogenesis, thermogenesis, nutrients uptake, and the adipose microenvironment. Furthermore, adipokines secreted into the circulation contribute to the regulation of various aspects, including appetite, cognitive function, energy expenditure, insulin secretion and sensitivity, endothelial function, and exercise capacity in the distant tissues. Ultimately, they modulate systemic metabolic homeostasis. The specific functions of certain adipokines are primarily determined by the distribution of their receptors and subsequent signaling pathways. For example, the anorexigenic effects of leptin on appetite regulation are mediated through LEPR and STAT3 signaling in specific neurons [22]. In the liver, ErbB receptors are responsible for harnessing NRG4 to exert an anti-lipogenic influence. Some adipokines utilize distinct receptors for various functions [105]. Asprosin, for instance, engages the OLFR734 receptor in the liver to enhance hepatic glucose production [69], while it employs Ptprd in the brain for its orexigenic actions [70]. Furthermore, different EVs display a diversity of target tissues. The specific uptake by these tissues is facilitated through the recognition of particular proteins in EVs by their respective receptors within the target tissues [203]. As a result, the identification of the target tissues and receptors of adipokines is crucial for comprehending their function and the underlying mechanisms.

The secretion of these adipokines is highly dependent on the energy and disease states of adipose tissues, such as fasting, cold exposure, exercise, aging, and obesity. However, it remains a challenge to specify the exact contributions of particular adipose tissue depots and their respective cell populations to the overall production of many adipose tissue-derived factors. Particularly, there is limited understanding of the differences in the adipokines secreted by visceral fat and subcutaneous fat. A recent study has shed light on this issue by demonstrating that visceral fat has increased secretory output compared with subcutaneous fat, secreting greater levels of chemokines, prostanoids, and extracellular matrix components [204]. These adipokines from visceral fat activate inflammatory signaling and disrupt insulin sensitivity in metabolic tissues, providing an explanation for the metabolic unhealthy effect of visceral fat. Additionally, in cases where several adipokines are also expressed in other organs, such as FGF21, chemerin, and BMPs, their endocrine contributions often remain undefined. One approach to address this issue involves the use of *in vivo* technologies based on arteriovenous adipokine analysis or microdialysis sampling, allowing for the quantification of secretions from adipose tissues. The use of fat-free mice and cell type-specific knockout or overexpression of these adipokines can also help in understanding their endocrine contributions.

Adipokines hold promise as candidates both for novel pharmacological treatment strategies and as diagnostic tools. For example, FGF21 analogs and agonists of FGFR1/KLB receptor complexes have displayed therapeutic potential in improving obesity and its associated complications. BFKB8488A (also bFKB1) is the bispecific agonist antibody for FGFR1 and KLB, which effectively emulates FGF21’s functions in both monkeys and humans [205]. In obese monkeys, BFKB8488A significantly reduced body weight and increased FGFR1 target gene expression in adipose tissue. A human study showed that a single BFKB8488A dose led to a brief decrease in body weight, ongoing improvements in cardiometabolic health, and a tendency to prefer less sweet and carbohydrate-rich foods [205]. These findings endorse the continued clinical exploration of this antibody-based therapy as a potential transformative treatment for metabolic abnormalities associated with obesity. Although significant progress has been made in recent years to advance adipokines like leptin, FGF21, and adiponectin as potential therapeutics into clinical studies, the paths to drug discovery pose great challenges. One major hurdle is the presence of resistance in individuals with obesity, such as leptin resistance and FGF21 resistance, which reduces responsiveness to the metabolically favorable actions of these adipokines. In addition, as many adipokines exert pleiotropic functions, sustained treatment increases the risk of adverse effects. For instance, the administration of NRG4 may yield beneficial cardiometabolic effects but simultaneously increase the risk of breast cancer development through prolonged activation of the receptors ErbB3 and ErbB4 [206]. Moreover, the development of oral drugs for these peptide factors is challenging, and thus pharmaceutical efforts to develop endocrine therapies based on these molecules are limited so far. Nevertheless, many adipokines exhibit beneficial effect on systemic metabolism in rodent models and non-human primates. The key is to transition the proof of concept derived from animal models to clinical settings. Demonstrating the feasibility and effectiveness of therapies based on the endocrine signaling of adipose tissue hormones in humans is of paramount importance.
